# The role of market in motivating farmers to reduce pesticide use: Evidence from vegetable farms in Shiraz

**DOI:** 10.1016/j.heliyon.2024.e35055

**Published:** 2024-07-23

**Authors:** Sara Larki Bolfarici, Mansour Zibaei, Dorna Jahangirpour

**Affiliations:** Agricultural Economics, Agricultural College, Shiraz University, Shiraz, Iran

**Keywords:** Environmental impact, Farmers' behavior, Pesticide overuse, Market incentives, Vegetable production

## Abstract

The overuse of pesticides has harmful impacts on both the environment and human health. Implementing efficient techniques is crucial to manage pesticides and reduce negative impacts effectively. In order to achieve this objective, we evaluated the harm of pesticide application in vegetable fields in Shiraz and identified the factors that impact farmers' behavior in using pesticides. The Environmental Impact Quotient (EIQ) indicator was initially utilized to evaluate the effects of pesticide residues on both human health and the environment. Afterwards, the Logit model was employed to investigate the likelihood of excessive pesticide usage among farmers. Our findings suggest that farmers may not give high importance to environmental and human health considerations when deciding on pesticide usage. Market incentives, such as the quantity and price of vegetables produced without pesticide use, play a significant role in reducing pesticide use in Shiraz vegetable farms. The results of the study can help policy makers in implementing pesticide-free agricultural practices.

## 1 Introduction

Vegetables are an important part of a healthy diet as they provide key nutrients that are crucial to human health [1]. Regular and daily consumption of vegetables in sufficient quantities prevents cardiovascular diseases, strokes, and certain cancers [2]. Hence, there are international programs to promote the consumption of vegetables in many countries [3]. The spread of disease occurs through fresh products [4]. Due to the substantial connection between vegetable consumption and human health, it is of the utmost importance to pay attention to the quality and health of the vegetables.

Proper application of pesticides is a key factor in enhancing the health of vegetables [1]. Moreover, the overutilization or repetitive application of pesticides poses a notable threat to both human health and the environment, in addition to effectively eradicating pests [5,6,7,8]. During recent decades, different indices have been used to assess the disadvantages of pesticides on the environment and humans in different countries [9,10,11,12].

The Environmental Impact Quotient (EIQ) is an index to assess the environmental disadvantages, which indicates the average harm of consumed pesticides such as potential risks to the health of farm workers, potential risks to consumers from pesticide residues on food and in groundwater, and adverse effects on the environment, including aquatic and terrestrial ecosystems. Many studies have employed this index to quantify and compare the effects of pesticides on different components. Some researchers stated that fungicides have the most negative effects on the environment [9,13]. [14] used EIQ to quantify the environmental impacts of Iranian wheat agroecosystem herbicides. A 71 % rise in herbicide use in wheat agroecosystems suggests that herbicides raised the overall risk. Their findings also show that difenzoquat are high-risk herbicide with high EIQ [15]. reported that fungicides have a significant negative impact on consumers in Iran. Carbendazim is an example of a harmful fungicide that poses risks to both farm workers and consumers and has the potential to leach into the Groundwater and lead to soil salinity [16]. [17] indicates that Imidacloprid (Confidor) is the most harmful insecticide in terms of its impact on the environment, farm workers, and consumers. Furthermore, the study revealed that insecticides like diazinon, malathion, and phosmet significantly harm the environment [16]. found that Diazinon insecticide causes the largest ecological damage in wheat and barley fields and Disinfectants have lower EIQs than paraquat herbicide in Mashhad wheat and barley farms.‏ [18] believe that in canola fields, Trifluralin herbicide is highly toxic to non-target biotas such as fish and algae. Therefore, the findings reveal that environmental degradation and biodiversity loss induced by pesticide overuse endangers agricultural product quality and safety, as well as food security [19,20].

Due to the adverse impact of excessive pesticide usage on both human health and the environment, efficient and effective policies should be designed and implemented to manage the use of pesticides. This goal can be achieved by identifying the factors affecting the behaviour of farmers in using pesticides. Some scientists suggest that inappropriate and excessive pesticide use is related to farmers' lack of knowledge about pesticide dosage and application methods [21,22,23], the strong dependence of crop production on the use of pesticides [24,21,25]. Lack of proper access to training [21] and farmers' low knowledge of the dangers caused by the excessive use of pesticides [26,27]. In addition, some research showed that the size of the family, the type of farm ownership [28,29], and the experience of farmers [30] affect the behaviour of farmers in determining the application rate of the pesticide [31]. state that farmers' pesticide safety behaviour can be improved by expanding their knowledge and changing their attitudes and perspectives. The information that farmers obtain and the degree to which they trust the information sources are the major factors that influence the farmers' decisions on the usage of pesticides. Farmers often rely on the advice of pesticide dealers, instructions on pesticide bottles, television and radio programs, and extension workshops for information on safe pesticide use [32,21,25]. Pesticide bottles contain accurate directions on pesticide use dose, spraying times, harvesting intervals, and pesticide container storage and disposal. According to Ref. [32], farmers don't use this information adequately, and it doesn't raise farmers' awareness about pesticide overuse [33]. Possible reasons for the aforementioned finding include the fact that farmers that are loss-averse may still use more pesticides in order to reduce the risk of income loss that may be brought on by disease and insect pests and maintain their own income stability [34].

Another body of literature indicates that technology, government regulations, and economics are three factors that influence the selection of the type and rate of pesticide usage [35,36,21,37,38]. Enhancing farmers' accessibility to innovative technologies and providing them with adequate training in utilizing such technologies [36], alongside promoting alternative methods to pesticides [39], have the potential to mitigate the dependence on pesticides and alleviate associated risks to human health and the environment. Economic factors such as income earned outside the farm, market revenue, and size of farms [29] influence the application rate of pesticides [40]. emphasize that market revenue incentives are more effective than government regulation on the behaviour of pesticide application of farmers. Although the findings indicate that market incentives play a more important role in determining farmers' pesticide use behaviour and have the potential to develop effective policies for pesticide usage reduction, it has received less attention in earlier studies.

The present study was conducted to identify the factors influencing farmers' behaviour in pesticide use. Since market incentives are one of the important factors affecting farmers' motivations and decisions in using pesticides, this study determines the role of price factors and farmers’ attitudes in using pesticides in the vegetable farms of Shiraz City in particular. Furthermore, since designing policies to deal with the excessive use of pesticides in vegetable farms is important, the policymaker should be aware of the seriousness of the problem. Hence, it is imperative to accurately assess and quantify the potential negative impacts associated with the use of pesticides on both human health and the environment. Thus, this research evaluates the environmental harmfulness of pesticides through the EIQ index.

## 2 Material and methods

**Fig. 1:**
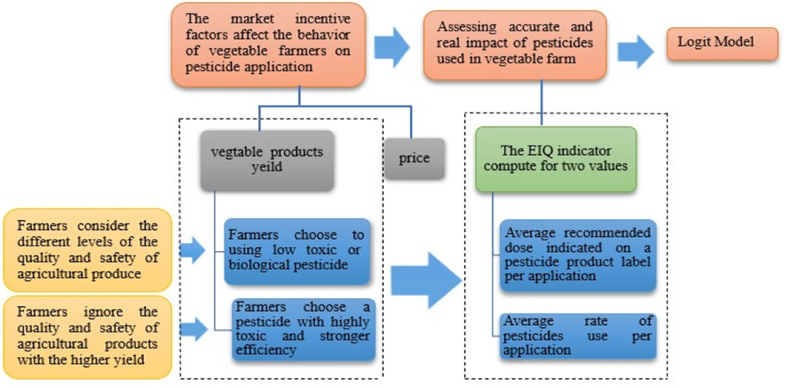
Examining the Role of market revenue on the Behavior of vegetable farmers on pesticide application.

The issues pertaining to the quality and safety of agricultural products are of significant concern, primarily attributed to the improper practices adopted by farmers. The decisions made by farmers regarding the quality and safety of agricultural products are influenced by the presence of information asymmetry. This study aims to analyze the impact of market revenue on the pesticide application behaviour of vegetable farmers in the presence of asymmetric conditions. Market revenue is comprised of two main factors: the quantity effect and the price effect. The quantity effect pertains to the yield of vegetable products achieved without the utilization of pesticides. On the other hand, the price effect denotes the price increase that occurs when emphasis is placed on the quality and safety of vegetable products. In this regard, two scenarios are considered. In the first scenario, it is assumed that the quality and health of the products are important for farmers. Therefore, they choose to use pesticides with less toxicity, or biological methods to manage pests and diseases with higher costs. In the second one, it is assumed that farmers ignore the quality and safety of vegetables, and choose a strong pesticide with higher yield and lower cost. The Logit model has been used in a number of earlier research to determine the variables impacting farmers' decisions about the rate of pesticide application [29,40]. In line with these findings, we used the Logit model to investigate the role of the market in motivating farmers to reduce pesticide use. The Logit model is a binary regression that aims to estimate the probability that the price and quantity of vegetables have an impact on farmers' decisions to reduce their use of pesticides. Fig. 1 shows the way to examine these scenarios briefly. The exact and real quantity of adverse effects of pesticides in vegetable farms was determined in the first step. In order to achieve this objective, the EIQ was computed based on the average pesticide consumption and the recommended dosage specified on the label of each pesticide product. In the subsequent phase, a comparison was made between the total EIQ in the administered dosage and the total EIQ in the recommended dosage. The role of price and yield on farmers' decision in choosing the type and rate of pesticide application by farmers was evaluated with the Logit model in the last step.

### 2.1 Study area

**Fig. 2:**
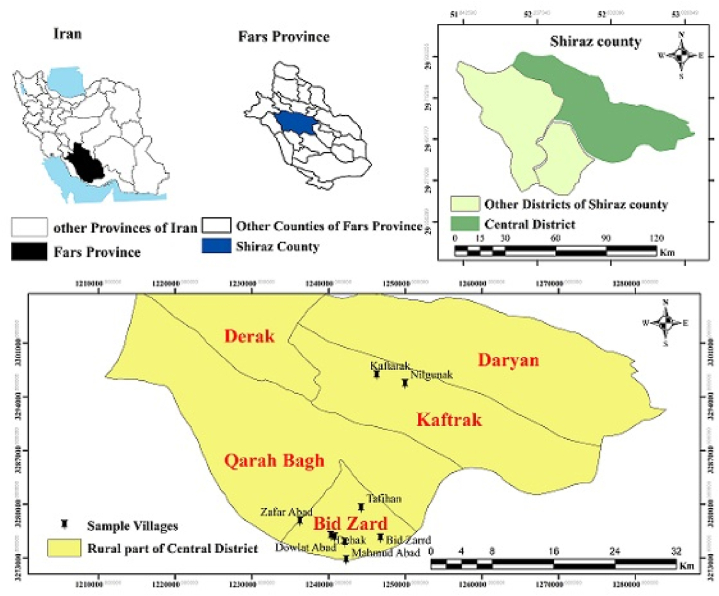
Geographical location of the study area.

This research was carried out in the villages of Shiraz County in Fars province (Fig. 2). Fars Province is located between the longitudes of 50° and 36 min to 55° and 35 min east, and the latitudes of 27° and 3 min to 31° and 40 min north. The area of this province is 122,607 square kilometers, and it has 29 cities based on the country divisions. The central district of Shiraz County has five rural regions including Bid Zard, Kaftarak, Qarah Bagh, Derak, and Daryan. This research was conducted in six villages of Bid Zard, Tafihan, Dowlat Abad Dehak, Esmail Abad, Zafar Abad, Mahmud Abad, as well as Kaftarak and Nilgunak, two districts of Bid Zard and Kaftarak villages, which are the districts of the central part. Based on the divisions, the study villages are located in the south and southeast of Shiraz. In the study areas, 12483 tons of vegetables are produced by the sample of farmers in this crop year. Other crops account for 21 % of vegetable cultivation.

### 2.2 Data collection

Between the years 2017 and 2018, a series of formal interviews and field observations were undertaken in order to collect data about farmers' pesticide use, their perception of pesticide risks, and general pest management practices to provide more detail. this survey focused on the pesticides used in farms cultivating lettuce, dill, coriander, leek, savory, parsley, tarragon, mint, spinach, and basil. Data were collected through face-to-face interviews with 164 farmers using a multi-stage random sampling method. First, a list of villages in Shiraz and the central region that cultivate vegetables was prepared. Then, farmers were randomly selected from each village and asked to complete the required information on the questionnaire.

For each crop, we asked respondents to list their worst pest concerns and how they managed them. Pesticide users were requested to provide the common name, the number of times sprayed, and the application rate. We asked respondents to exhibit the bottle (if feasible) or describe its appearance or use if they couldn't remember the popular name. The shops and specialists affiliated with the Plant Conservation Organization of Agriculture Jihad Fars have provided comprehensive data on the active ingredients of each pesticide. We classified pesticides according to their active ingredients, grouping them into three categories based on their purpose: insecticide, fungicide, and herbicide. We computed the weighted average of each pesticide application by taking into account the reported application rates provided by the farmers on the sample farms. Subsequently, we employed the EIQ indicator to assess the level of toxicity generated by pesticides at both the average dose and the recommended dose specified on the pesticide product label. This indicator has the benefit of being computable, even in cases where information is limited. We conducted an assessment of the toxicity caused by pesticides at both the average dose and the recommended amount specified on the pesticide product label. This assessment was carried out on various vegetables produced in sample farms, including lettuce, dill, coriander, basil, mint, savory, spinach, parsley, Tareh Irani, and Tarragon farms. In addition, we assessed the toxicity caused by pesticides at both the average and recommended levels of a pesticide product, as indicated by 164 farmers who provided samples. Ultimately, we applied the Wilcoxon test to evaluate the toxicity resulting from the rate of pesticide application and the recommended pesticide dose, with the goal of investigating the tendency of farmers to apply pesticides at the recommended amount.

### 2.3 The quantification of pesticide overuse

The EIQ method is a comprehensive measure of environmental impacts associated with the use of pesticides. It serves to assess and quantify the potential hazards posed by specific pesticides. The EIQ value of an individual pesticide is the average of the farm work, consumer, and ecological components, which is calculated as follows:

(1) $$EIQ=\left({EIQ}_{\text{Farmworker}\;\text{Component}}+{EIQ}_{\text{Consumer}\;\text{Component}}+{EIQ}_{Ecological\;\text{Component}}\right)/3$$

Based on this model, the environmental effects can be compared between pesticides and different pest management programs [41], and finally the appropriate pest control programs are selected. The EIQ field use rating was determined by using the weighted average application rate of each pesticide and the prescribed dose from the product label to make crop-to-crop comparisons of environmental impact as follows:

(2) $${\text{EIQ}}_{\text{Field}\;\text{Use}\;\text{Rating}}=EIQ\times A\times active$$

A denotes the weighted average application rate/recommended dose (expressed in kg/ha) of a pesticide when applied each time. The variable "active" represents the proportion of active ingredients in the pesticide, while EIQ refers to the average EIQ base value of these active ingredients. The formula consequently calculates the cumulative EIQ by aggregating the ratings of the pesticides employed in crop cultivation, thereby facilitating the determination of EIQ baseline values. It is assumed that the farmers in the sample farms use the pesticides based on the recommended dose. Therefore, the non-parametric Wilcoxon test was used to test the hypothesis, by which the toxicity caused by the application rate of pesticides is equal to the toxicity caused by the recommended doses. In this test, the difference between the toxicity caused by the application rate of pesticide and the toxicity caused by the recommended dose is ranked. Then, the Z statistic for testing the hypothesis is calculated as follows [42]:

(4) $$Z=\frac{\frac{T-\left(n\left(n+1\right)\right)}{4}}{\sqrt{\frac{n\left(n+1\right)\left(2n+1\right)}{24}}}$$

where *T* is the sum of the toxicity rankings, which are created by the application rate of pesticide in the sample of farms more or less than the recommended doses, and *n* is the total sample size.

### 2.4 Farmers' overuse behaviour

Logistic regression was employed to analyze the relationship between independent variables and a binary dependent variable, which represents the probability of pesticide overuse behaviour among farmers. This approach was utilized to identify the factors influencing such behaviour [43]. The logit model follows the logistic distribution. The logistic distribution is always zero and one and is defined as follows:

(5) $$f\left(z\right)=\frac{e^{z}}{1+e^{z}}$$

The logistic function ranges from inordinate negative to inordinate positive, while the outputs of the logistic function are between zero and one [43]. The logistic function involves the utilization of the Z variable, which represents a collection of independent variables. Meanwhile, f(z) denotes the probability associated with a specific outcome, given the aforementioned set of explanatory variables. The variable Z is commonly designated as:

(6) $$z=\beta_{0}+\sum_{m=1}^{K}\beta_{m}x_{m}$$

where $\beta_{0}$ is the intercept, and $\beta_{1}$ to $\beta_{m}$ are the coefficients regression of $x_{1},x_{2},\ldots .,x_{m}$ respectively. The dependent variable, pesticide overuse behaviour, is characterized by binary values. It is defined as 1 if the sum of the EIQ in the application rate of pesticides is smaller than the sum of EIQ in the recommended doses, and 0 otherwise. The expression of pesticide overuse behaviour can be represented as a function of various independent variables corresponding with the farmer. These variables are collected and organized in a vector denoted as x, such that:

(7) Overusebehavior(dummy)=f(price,quantity,land,ownership,risk,age,education,familysize,job,income,time)

The probability that farmers use pesticides more than the recommended dose is equal to:

(8) $$P\left(\text{Overuse}\;\text{behavior}=1|X_{i}^{\prime }\right)=G\left(X_{i}^{\prime }\beta \right)=\frac{1}{1+e^{-X_{i}^{\prime }\beta }}$$

Table 1 describes the independent variables.

**Table 1:** Descriptive statistics of the independent variables included in the logit model.

Variable	Description	Mean	SD	Minimum	Maximum
Quantity effect ((quantity)	The possible reduction of the product from the point of view of the sample of farmers (in percentage)	71.96	28.47	10	100
Price effect ((price)	The price increases when paying attention to the quality and safety	78.12	23.12	25	100
Age ((age))	Age of respondent (years)	45.75	14.26	21	85
Education ((education))	Education level	–	–	1	5
Family size (familysize)	Dependent people in the family	1.90	1.33	0	6
Risk perception (risk)	Perceptions of pesticide risk (mean index of risk). The average of ranks is considered.	–	–	1	5
Income ((income)	The logarithm of annual income	5.62	0.39	4.47	6.39
Earn money from a job outside the farm ((off−farm))	Earning money from a job outside the farm	3.56	1.49	1	5
Land area ((land)	Land area (hectares)	4.07	5.38	1	50
Land ownership (ownership)	Land ownership(ordinal)	–	–	1	2

## 3 Results

### 3.1 Socioeconomic characteristics of interviewee farmers

The data presented in Table 1 display the socioeconomic characteristics of the interviewed farmers. Within the surveyed region, the mean age of farmers was determined to be 45.75 years, with a majority of 64.6 % falling within the age range of 20–50 years. A significant number of respondents (23.8 %) were illiterate, 26.8 % had not completed primary and secondary education, and 15.9 % had completed secondary education. 27.6 % had finished high school, and 6.1 % had higher education. 53.7 % of farmers have more than 20 years of agricultural experience. farmers possess 669.03 ha of agricultural land, of which about 426 ha are cultivated with lettuce and leafy vegetables such as dill, coriander, beetroot, spinach, basil, mint, leek, savory, tarragon, watercress. The percentage of 63.64 of the sample of vegetable farmers were the owners of their vegetable farms. The mean income of farmers in the previous crop year (2017–2018) was about 33 million Rials. The income of 48.8 % of farmers is 100 % dependent on agriculture.

### 3.2 Respondents' sources of information on safe pesticide use

**Fig. 3:**
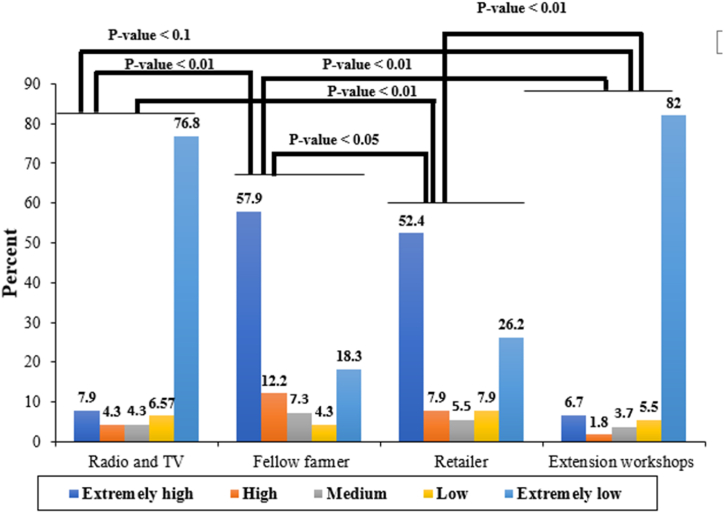
The share of different information sources in farmers' knowledge acquisition about the safe use of pesticides P-value incidents the significance level of the comparison of pairs of answers regarding different information sources using the Wilcoxon test.

Farmer pesticide use is influenced by radio and TV educational programs, fellow farmers, pesticide sellers, and Extension programs. Farmers' trust in different sources of pesticide safety information is measured on a 5-point Likert scale (extremely high = 5, high = 4, medium = 3, low = 2, and extremely low = 1). The interviewees' answers about sources of information on safe pesticide use have been analysed in pairs using the Wilcoxon test. The results indicate that there is a significant difference between the reliance on advice from pesticide retailers and fellow farmers compared to information obtained from radio and television, and extension workshops. This incident that farmers rely more likely on the advice of pesticide retailers and fellow farmers compared to information obtained from radio and television, and extension workshops. Neither radio nor television instructional programs are particularly effective at disseminating knowledge about safe pesticide usage, and there aren't many farmers who actually heed the advice they give (Fig. 3).

Another important source for the sharing of agricultural information is from other farmers. The results show that over 12 % of farmers obtain a significant portion of their information from their fellow farmers, while more than half of farmers (57.9 %) rely on safe pesticide usage advice from their peers. About 7.3 % of farmers stated using information from fellow farmers sometimes, while approximately 4.3 % of farmers reported receiving a low amount of information from their peers. On the other hand, roughly 18 % of farmers reported receiving extremely low levels of information from their fellow farmers (Fig. 3).

The data reveal that about half of farmers rely on advice about safe pesticide use provided by pesticide retailers (52.4 %), indicating that farmers base their decisions on the advice of retailers. Additionally, 7.9 % of farmers follow even more information from pesticide retailers. About 5 % of farmers receive and follow information from pesticide retailers, while 7 % of farmers use only a small portion of the information provided by pesticide retailers. Furthermore, almost a quarter of farmers rarely rely on recommendations from pesticide retailers (26.6 %). (Fig. 3).

The data Based on the data collected in the questionnaires, it was found that only a small percentage of farmers rely on extension officers to acquire knowledge about pest control and make decisions about pesticide use. Additionally, a very small proportion of farmers primarily obtain information on the safe use of pesticides from extension services, accounting for only 1.3 % of the total. Almost 3 % of farmers sometimes receive and follow advice from extension officers. In contrast, 5.5 % of farmers receive and follow less information from extension officers, while more than three-quarters of farmers rarely trust extension officers' advice on safe pesticide use (82 %) (Fig. 3).

### 3.3 Farmers' perceptions of risk associated with pesticide usage

The indiscriminate use of pesticides, caused by farmers' misuse of pesticide application, poses risks to human health and the environment. This behaviour stems from a lack of awareness of the risks that pesticides pose to human health and the environment. The level of perceived risk associated with pesticides was measured in three sections, with each statement evaluated on a 5-point Likert-type scale (ranging from 1 to 5) as follows: 1 = extremely small risk, 2 = small risk, 3 = medium risk, 4 = high risk, and 5 = extremely high risk. The first part evaluated farmers' awareness of the dangers of pesticides to ecological components, while the second part evaluated farmers' awareness of the dangers that pesticides pose directly and indirectly to human health. The third part evaluated farmers' awareness of the dangers of pesticides and their willingness to produce healthy vegetables.

**Fig. 4:**
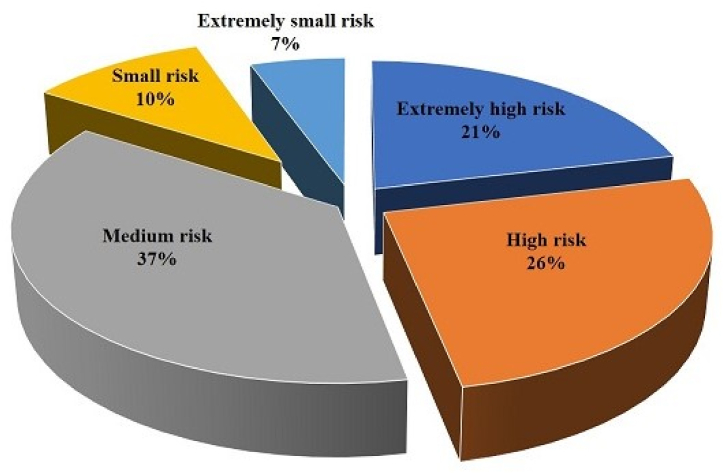
Farmers' perceptions of risk from pesticides.

Approximately 21 % of farmers asserted that pesticide residues pose an extremely high risk, while 26 % indicated that pesticides present a high level of risk to both human health and the environment. A notable proportion, specifically 37 %, of the farmers exhibited awareness regarding the detrimental consequences associated with the utilization of pesticides, thereby expressing a desire to cultivate vegetables that promote health and well-being. In contrast, approximately 10 % of farmers expressed the viewpoint that pesticides have minimal negative impacts on both human health and the environment, whereas 7 % of farmers asserted that pesticides have indeed had extremely small risks on human health and the environment. The figure presented in Fig. 4 illustrates the risk perceptions of farmers towards pesticides.

### 3.4 The quantification of pesticide overuse

**Fig. 5:**
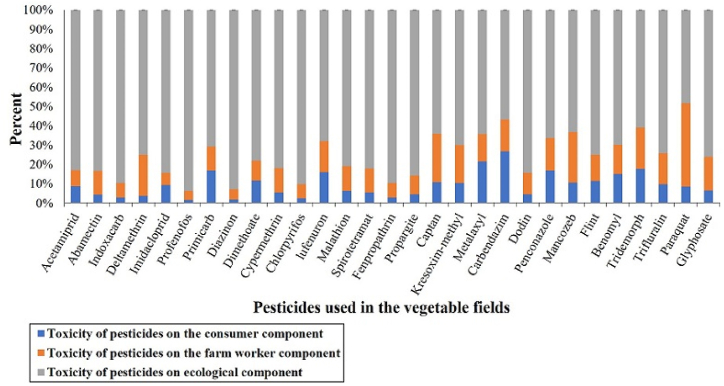
The assessment impact of pesticides by the EIQ indicator for a farm worker, a consumer, and ecological components in vegetable farms.

The studied farmers reported thirty-two types of permitted pesticides in the three groups of insecticides, herbicides, and fungicides used to annihilate pests and control diseases and weeds. Fig. 5 plots the impact of pesticide residuals via the EIQ indicator on three components in vegetable farms: farm worker, consumer, and ecological. Fig. 5 shows that pesticide residuals endanger human health and harm the environment. Environmental damage is higher than human health risks, in which case, the possibility of destroying the environment and reducing biodiversity increases over the years.

**Fig. 6:**
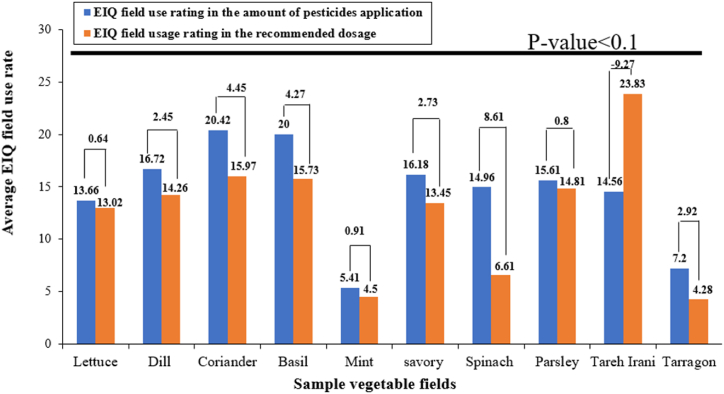
The average environmental impact on vegetable farms P-value incidents the significance level of the comparison of EIQ field rating in the amount of pesticides application and EIQ field rating in recommended dosage in vegetable farms using the Wilcoxon test.

On average, in each cycle of crop cultivation, pesticides are applied 11.8 times, with a median of 8 times. Fig. 6 shows the EIQ in vegetable farms. The Wilcoxon test indicates a significant difference between EIQ field rating in the amount of pesticides application and EIQ field rating in recommended dosage in vegetable farms. The EIQ based on the dose of pesticides used by vegetable farmers in the fields of dill, coriander, basil, savory, spinach, and tarragon is higher than the EIQ based on the recommended dose, resulting in lower quality and safety in production and marketing. The toxicity caused by the pesticides used in the fields of lettuce, mint, and parsley is not much different from the ones caused by using pesticides in the recommended dose. Therefore, it is not possible to comment on the quality and safety of these products. This slight difference may be caused by the incorrect answers of the farmers about the application rate of pesticides, miscalculations, and the lack of appropriate management.

The Wilcoxon signed rank test shows the tendency of farmers to produce safe and high-quality vegetables (Table 2). This test compares the toxicity caused by the application rate of the pesticide with the toxicity caused by the recommended dose. The Z-statistic is 7.978. Based on this statistic, farmers do not have the same tendency to produce safe and high-quality vegetables. In 29 vegetable farms, the toxicity level of pesticide application was set lower than the recommended dose, which means that farmers applied pesticides at lower than the recommended dose, while in 7 vegetable farms, the toxicity level of pesticide application was set as many times as the recommended dose, which means that farmers applied pesticides at the recommended dose according to the instructions. However, in most of the vegetable farms, the toxicity of the applied pesticides was higher than the recommended dose, indicating that farmers chose to apply pesticides more than the recommended dose (in 128 vegetable farms). Farmers' behaviour could be explained by the fact that they do not read the directions printed on pesticide bottles and do not rely on agricultural extension agencies; instead, they rely on their fellow farmers and retailers. As a result, the vegetables grown on these farms are of inferior quality and are sold on the market.

**Table 2:** Evaluating the willingness of farmers to produce quality and safety vegetables.

	Number	Average ranks
Toxicity caused by the recommended dose indicated on a pesticide product label > Toxicity caused by the rate of pesticides applied.	29	56.88
Toxicity caused by the recommended dose indicated on a pesticide product label < Toxicity caused by the rate of pesticides applied.	128	84.01
Toxicity caused by the recommended dose indicated on a pesticide product label = Toxicity caused by the rate of pesticides applied.	7	-a
Total	164	Z = −7.978

a This test does not report the average rank for this assumption.

### 3.5 Farmers' overuse behaviour

The findings of Logit regression analysis indicate that variables such as education, family size, income, off-farm activities, land ownership, price, quantity, and time exhibit a statistically significant impact on farmers' pesticide application behaviour. pseudo $R^{2}$ measures (0.9168), log-likelihood statistics (−8.357), and LR $x^{2}$ (209.76) indicate goodness of fit of the Logit model (Table 3). Table 3 shows that a higher level of education, bigger families, higher prices of vegetables and higher income decrease pesticide use in a statistically significant manner. The opinion is that the decrease in yield is due to raising pesticide use higher than the recommended dose in vegetable farms. Farmers rely more on pesticides in large vegetable farms and use pesticides higher than the recommended dose. Farmers who earn money from a job outside the farm use pesticides higher than the recommended dose. Farmers raise the rate of application of pesticides higher than the recommended dose by increasing spraying times.

**Table 3:** Logit regression results for pesticide overuse behaviour among farmer.

Independent variable	Coefficient	Standard error	Z	Marginal effect
quantity	0.23	0.09	2.5**	0.003
price	−0.3	0.12	−2.4**	−0.004
age	−0.15	0.1	−1.55	−0.002
education	−3.77	1.41	−2.67***	−0.059
familysize	−3.98	0.81	−2.19**	−0.062
risk	−0.46	1.85	−0.79	−0.022
income	−6.24	3.3	−1.89*	−0.09
off−farm	11.01	5.4	2.04**	0.172
land	0.55	0.24	2.25**	0.008
ownership	−2.83	2.3	−1.23	−0.044
Time	0.09	0.04	2.12**	0.001
_cons	21.69	15.24	1.42	–

## 4 Discussion

The results of this study indicate that most farmers in Shiraz rely heavily on pesticides to kill weeds and pests and control diseases. Most of the farmers tend to overuse pesticides which leads to an increased toxicity of the applied pesticides in most vegetable farms. The results confirm previous studies indicating the overuse of pesticides in Iran. Analysis of vegetable residues in local markets also indicates such a result [44,45]. Our results suggest that the excessive use of pesticides harms the ecological component. This result is consistent with the study of [15] in barley and wheat fields of Mashhad, which indicates the necessity to develop effective and urgent policies to manage pesticide use in vegetable fields.

Farmers rely on the advice of fellow farmers and retailers regarding information on safe pesticide use. Our results show that trust in fellow farmers is rather high. Several prior studies have demonstrated that farmers depend on one another for the purpose of pesticide selection as well as determining appropriate pesticide dosages [30,29,46]. [47] reported that trust in pesticide retailers plays a critical role in transferring information to farmers and increases the likelihood that they will increase the recommended dose of pesticide use. These results are consistent with previous studies conducted by Refs. [48,49,30,29,50,46]. These studies have reported that most farmers get their information regarding pesticide application from retailers in other countries, Pesticide retailers are promoting an aggressive use of pesticides pesticide usage among farmers [ 3, 22, 41]. This promotion is primarily driven by the profit motive, as higher sales directly translate to greater profits for these retailers [48]. Extension workshops are a cost-effective and efficient approach to promoting behavioural change among farmers concerning pesticide reduction. Engagement in extension programs enhances farmers' understanding and consciousness regarding the adverse consequences of excessive pesticide utilization and alternative pest management approaches [30,25,51]. According to the findings of [30], the implementation of extension workshops resulted in a decrease in the utilization of pesticides. Our result shows extension officers’ advice has no significant influence on pesticide application behaviour. These results are consistent with [46]. Radio and TV are important sources of information regarding safe pesticide use. Our results show that Farmers do not pay attention to their advice. These findings concur with those of [29].

The education level of farmers, which was considered in order from illiterate to higher education, has a negative effect on the behaviour of farmers' pesticide application. By increasing the educational level of farmers, the possibility of using pesticides decreases by 0.06. Larger households care more about food safety and quality. On average, with the increase of one person in the household, the possibility of using more than the allowed limit of pesticides in vegetable fields decreases by 0.08. Our analysis revealed that the variable land has a positive effect on farmers' tendency to overuse pesticides, with an increase of 0.008 in the probability of pesticide use for each additional hectare of land. In other words, farmers with larger farms are more concerned about maintaining a stable income from their farms and may tend to overuse pesticides in vegetable production to achieve this. These findings are consistent with that of [40]. Farm owners are assumed to use fewer pesticides because the overuse of pesticides reduces efficiency and land value over time [28]. However, the type of farm ownership does not significantly affect pesticide use, which could be related to farmers' income dependence on agriculture. The variable off-farm income, which represents the degree of dependence of the farmer's livelihood on agriculture, indicates that farmers who are more dependent on income from vegetable production tend to overuse pesticides. On the other hand, the level of farm income has a significant effect on the tendency to overuse pesticides. The effect of off-farm income is larger than that of farm income. Consequently, farmers who are dependent on the farm and have no other source of income use more pesticides to control pests and diseases to achieve reliable crop yield.

Farmers increase the rate of pesticide application by increasing the number of sprays on vegetable crops. The logit model results reveal that spraying additional pesticides raises the likelihood of pesticide use by 0.001. It could be for the reason stated by Ref. [52]. They say that farmers believe that more spraying is needed to prevent pests and illnesses from reducing output or profitability.

The results of our study indicate that although farmers are aware of the potential risks associated with pesticide use, the variable risk has a non-significant effect on their pesticide use behaviour. This suggests that farmers may not prioritise environmental and human health concerns when making decisions about pesticide use (as shown in the logit table). This finding aligns with the research conducted by Ref. [30], which similarly concluded that farmers in developing countries might not use pesticides in a manner that effectively mitigates potential risks to both human health and the environment.

Market incentives, such as the quantity and price of vegetables produced in the absence of pesticide use, play a major role in reducing pesticide use on vegetable farms. There is a positive relationship between quantity and the tendency of farmers to overuse pesticides. In this case, the probability of using more than the recommended dose of pesticides increases by 0.03. There is a negative relationship between price and the tendency of farmers to overuse pesticides. If the price increases by 1 %, the probability of excessive use of pesticides decreases by 0.04. Therefore, farmers are of the opinion that the decrease in yield resulting from not using pesticides can be balanced out by the increase in vegetable prices. Consequently, they reduce pesticide use and produce high-quality vegetables in large quantities. Regarding the influence of market factors (price and quantity), the findings are consistent with those of [28,40].

## 5 Conclusion and recommendations

Although it is necessary to reduce pesticide use in order to improve agricultural sustainability and protect the environment and human health, farmers are less likely to adopt alternative pest management strategies because they believe it will result in crop yield and income losses. Consequently, it is necessary to investigate the influencing factors on farmers' behaviour regarding pesticide excessive use to design appropriate strategies for pesticide use reduction in vegetable farms.

The present study focused on the factors influencing Shiraz farmers' decisions regarding pesticide use. The findings demonstrated that the pattern of pesticide use in Shiraz vegetable fields seriously endangered the health of farmers and consumers of vegetables as well as the quality of the environment. It is challenging to research the effects of pesticides on farmers' behaviour in Iran because there are no strong laws and controls on their use. According to the results, high-quality products are generated when consumers pay attention to quality and the vegetable market mechanism, which can improve the quality and safety of the vegetables produced. As a result, the government can apply two strategies to encourage farmers to grow healthful vegetables. First, the government needs to take steps like yield insurance to allay farmers' concerns about their income. Second, policy makers need to make sure that healthy, high-quality products are marketed, as this will help farmers who produce high-quality goods to get paid more in the future. Therefore, it is advised to create a framework for eliminating middlemen, increasing the price of products at farms, and creating a system for distributing commercial brands and quality certificates.

## CRediT authorship contribution statement

**Sara Larki Bolfarici:** Writing – original draft, Visualization, Validation, Software, Resources, Methodology, Investigation, Formal analysis, Data curation. **Mansour Zibaei:** Validation, Supervision, Methodology, Conceptualization. **Dorna Jahangirpour:** Writing – review & editing, Visualization, Validation, Software, Formal analysis.

## Declaration of competing interest

The authors declare that they have no known competing financial interests or personal relationships that could have appeared to influence the work reported in this paper.
