# Supplementary material for: The role of market in motivating farmers to reduce pesticide use: Evidence from vegetable farms in Shiraz
Source: Heliyon. 2024 Jul 23;10(15):e35055. doi: 10.1016/j.heliyon.2024.e35055 (PMC11332851; doi:10.1016/j.heliyon.2024.e35055)
Supplement: Multimedia component 1 [file mmc1.docx]

**The role of market in motivating farmers to reduce pesticide use: Evidence from vegetable farms in Shiraz**

**
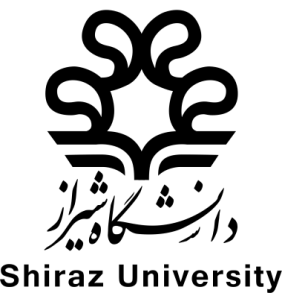
Questionnaire number: ………… Date: ……/……/……..**

| **The questionnaire is intended to do a research project. Your name and personal information will not be recorded in this questionnaire. The aim of the research is to investigate the factors affecting the behavior of farmers regarding use of pesticides in vegetable farm. You are selected based on the completely random sampling method. The information shared with us will be shown in the form of a general statistic and will be held in strict confidence.** |
| --- |

**A. Profile of farmers**

| 1.Name and surname: ……………….. 2.Rural district: ………………….  3. Village: ……………. 4. Age: ………. Years old  5. Sex: …………. 6. Education level: …………  7. Marital status: Single Married  8. Dependent people in the family: …………...  9. How many years have you been farming?  I have been farming between one and five years.  I have been farming between six and ten years.  I have been farming between 11 and 15 years.  I have been farming more than 20 years  10. Do you own the land? Yes, I own the Land. No, I rented the land.  11. How much do you have land farming? I have …….... hectares land farming.  12. How much do you have vegetable farm? I have ………. hectares vegetable farm.  13. What kind of vegetables do you grow? ……………………..  14. How many times a year do you plant vegetables? …………….. a year.  15. How much did you earn last year? ……………….  16. How much does your livelihood depend on farming? It means, do you earn money from a job outside the farm?  I earn my entire income from farming.  I earn 80% of my income from farming.  I earn 50% of my income from farming.  I earn 30% of my income from farming.  I earn 10% of my income from farming.  17. what kind of method do you use to kill pests and management diseases?  I use pesticides to kill pest and management diseases.  I use biological methods. |
| --- |

**B. The number of pesticide applications in one crop season**

| 1.What is your criteria choose pesticides?  I choose pesticide strong that can kill all pests.  I choose pesticide weak that only kills pests and diseases that damage the farm.  2. How has the trend of pesticide use changed in the recent years?  The trend of pesticide use has not changed in the recent years.  The trend of pesticide use has reduced in the recent years.  The trend of pesticide use has risen in the recent years. |
| --- |

**C. The opinion of the farmers about the positive effects of using pesticides**

I request you express your opinion regarding the following statements.

|  | **Completely Agree** | **Agree** | **Have No Opinion** | **Disagree** | **Completely Disagree** |
| --- | --- | --- | --- | --- | --- |
| The use of pesticides increases production. |  |  |  |  |  |
| The use of pesticides leads to the elimination of pests and diseases. |  |  |  |  |  |

**D. Information regarding crop year 2017-2018**

| **Vegetable name** | **The number of planting times in a year** | **Planting date** | **Harvest date** | **The Name of pesticide used** | **The Dose of pesticide used in a hectare** | **Frequency** | **Amount of vegetable harvest** | **Price of vegetable** |
| --- | --- | --- | --- | --- | --- | --- | --- | --- |
|  |  |  |  |  |  |  |  |  |
|  |  |  |  |  |  |  |  |  |
|  |  |  |  |  |  |  |  |  |
|  |  |  |  |  |  |  |  |  |
|  |  |  |  |  |  |  |  |  |
|  |  |  |  |  |  |  |  |  |
|  |  |  |  |  |  |  |  |  |
|  |  |  |  |  |  |  |  |  |
|  |  |  |  |  |  |  |  |  |
|  |  |  |  |  |  |  |  |  |
|  |  |  |  |  |  |  |  |  |
|  |  |  |  |  |  |  |  |  |
|  |  |  |  |  |  |  |  |  |
|  |  |  |  |  |  |  |  |  |
|  |  |  |  |  |  |  |  |  |
|  |  |  |  |  |  |  |  |  |
|  |  |  |  |  |  |  |  |  |
|  |  |  |  |  |  |  |  |  |
|  |  |  |  |  |  |  |  |  |
|  |  |  |  |  |  |  |  |  |

**E. Farmers' perceptions of risk from pesticides.**

I request you express your opinion about the following statements.

|  | **Extremely High Risk** | **High Risk** | **Medium Risk** | **Small Risk** | **Extremely Small Risk** |
| --- | --- | --- | --- | --- | --- |
| Pesticides reduce fertile soil (ecological risk). |  |  |  |  |  |
| Pesticides create biological disruption in the environment (ecological risk). |  |  |  |  |  |
| Pesticides cause Groundwater pollution (human health risk). |  |  |  |  |  |
| Excessive pesticide application creates acute and chronic diseases such as Cancer and skin disease (human health risk). |  |  |  |  |  |
| The Product quality depends on the correct use of pesticides during the cultivation of vegetables (dangers of pesticides and willingness to produce healthy vegetables). |  |  |  |  |  |
| Farmers who are the biggest owner of farmland use more than pesticides (dangers of pesticides and willingness to produce healthy vegetables). |  |  |  |  |  |
| Quality and safety food and organic products are important (dangers of pesticides and willingness to produce healthy vegetables). |  |  |  |  |  |
| Soil and water are the source of life and must be carefully preserved (dangers of pesticides and willingness to produce healthy vegetables). |  |  |  |  |  |
| The key to agricultural success is knowing and protecting the environment (dangers of pesticides and willingness to produce healthy vegetables). |  |  |  |  |  |
| Pesticide application is the last and best way to control and manage pests and diseases (dangers of pesticides and willingness to produce healthy vegetables). |  |  |  |  |  |
| Successful farmer doesn’t use pesticide during the cultivation of products and produces quality and safety products (dangers of pesticides and willingness to produce healthy vegetables). |  |  |  |  |  |
| Use of weak pesticides and their repetition is better (dangers of pesticides and willingness to produce healthy vegetables). |  |  |  |  |  |
| Use of strong pesticides and their less repetition is better (dangers of pesticides and willingness to produce healthy vegetables). |  |  |  |  |  |

**F. Correct use of pesticide**

I request you express your opinion about the following statements. The statements show how it is important produce and supply safety and quantity vegetable.

|  | **Extremely High** | **High** | **Medium** | **Low** | **Extremely Low** |
| --- | --- | --- | --- | --- | --- |
| I use more than recommended dose on the label. |  |  |  |  |  |
| I Supply vegetable to the market shortly after spraying. |  |  |  |  |  |

**G. sources of information on safe pesticide**

How effective the following information sources are on the correct use of pesticides?

|  | **Extremely High** | **High** | **Medium** | **Low** | **Extremely Low** |
| --- | --- | --- | --- | --- | --- |
| I receive and follow the information regarding safe pesticide use from Tv and radio. |  |  |  |  |  |
| I receive and follow the information regarding safe pesticide use from pesticide retailers. |  |  |  |  |  |
| I receive and follow the information regarding safe pesticide use from fellow farmers. |  |  |  |  |  |
| I receive and follow the information regarding safe pesticide use from extension workshops. |  |  |  |  |  |

**I. Quantity effect**

| 1. If you don’t use pestisides to kill pests and management diseases, quantity production ………  reduces doesn’t change  -How much does production rate change as a persentage? ………………….. |
| --- |

**H. Price effect**

| - How much should the price of vegetables increase to avoid the use of pesticides and produce a vegetable without of pesticides?  The price of vegetable should increase from the current price ……….. Rials for a kilo of vegetable to ……. Rials for a kilo of vegetable. |
| --- |
